# Analysis of agreement between specialists for the evaluation of radiological findings of necrotizing enterocolitis

**DOI:** 10.1016/j.jped.2024.07.008

**Published:** 2024-08-21

**Authors:** Erica Cristina Scarpa, João C. Lyra, Pedro L.T. de A. Lourenção, Andréa S. Hachem, Geraldo H.S. da Silva, Glauce R.F. Giacóia, Erika V.P. Ortolan, Camila de Paula Silva, Guilherme L. da Silveira, Maria R. Bentlin

**Affiliations:** a Universidade Estadual Paulista (UNESP), Faculdade de Medicina de Botucatu, Departamento de Pediatria, Divisão de Neonatologia, São Paulo, SP, Brazil; b Universidade Estadual Paulista (UNESP), Faculdade de Medicina de Botucatu, Departamento de Cirurgia e Ortopedia - Divisão de Cirurgia Pediátrica, São Paulo, SP, Brazil; c Hospital Israelita Albert Einstein, São Paulo, SP, Brazil; d Mega Imagem Clinic, Santos, SP, Brazil

**Keywords:** Abdominal radiography, Concordance study, Necrotizing enterocolitis

## Abstract

**Objective:**

The analysis of abdominal radiography is essential for the diagnosis and management of necrotizing enterocolitis (NEC) in newborns (NB). Studies, however, show a lack of agreement among physicians in the interpretation of images. This study aims to evaluate the agreement in the radiological interpretation of the NEC between examiners from different specialties (interexaminer analysis) and between the same examiner at different times (intraexaminer analysis).

**Methods:**

Cross-sectional study for concordance analysis using plain radiographs of the abdomen of NB with suspected or confirmed NEC. The study included two neonatologists (Neo), two surgeons (SU), and two radiologists (RD). The participants filled out a form with questions about the radiographic findings; regarding the presence of intestinal loop distension, the specialists answered subjectively (yes or no) and objectively (calculation of the ratio between loop diameter and lumbar vertebrae measurements). Kappa coefficients were calculated for agreement analysis.

**Results:**

A total of 90 radiological images were analyzed. For the interexaminer evaluation, the agreement was low (kappa<0.4) in 30 % of the answers (Neo versus SU), 38 % (Neo versus RD), and 46 % (SU versus RD). In the intraexaminer evaluation, the neonatologist and the surgeon presented substantial or almost perfect agreement in 92 % of the answers, and the radiologist in 77 %. In the evaluation of intestinal loop distention, the greatest agreement between the specialties occurred when done objectively.

**Conclusion:**

The results confirmed the low intra- and interexaminer agreement in the radiological analysis of the NEC, reinforcing the importance of standardizing the methods of radiological interpretation of the disease.

## Introduction

Necrotizing enterocolitis (NEC) is a severe inflammatory disease of the gastrointestinal tract (GIT) typically found in preterm infants with very low birth weights (VLBW), especially in those younger than 28 weeks of gestational age (GA).^1^, ^2^, ^3^, ^4^ The incidence varies from 5 to 12 % of neonates born at a VLBW, and it increases as the GA and birth weight (BW) lowers.^5^, ^6^, ^7^, ^8^, ^9^, ^10^ The pathogenesis of NEC is multifactorial, and the clinical presentation is variable; its onset may be insidious, with nonspecific findings, or fulminant, in which it evolves rapidly into shock.^11^, ^12^, ^13^

The diagnosis of NEC is complex and challenging. If, on the one hand, the diagnosis can be late, in advanced stages, on the other hand, “overdiagnosis” of the disease is often observed, causing unnecessary measures, such as indication of prolonged fasting, use of antibiotics, and surgical intervention.^14^ Although the radiological examination of the abdomen, in association with clinical findings, is an important tool for the diagnosis of the disease, divergences regarding the evaluation often occur among the professionals involved in the care, which can result in inadequate conduct, bringing consequences for the prognosis of the newborn (NB).^15^ Therefore, studies that evaluate the agreement between physicians involved in the management of NEC are useful to identify the main points of disagreement in the interpretation of the radiological findings, typical of NEC, and thus facilitate the elaboration of systematized protocols for this evaluation.^15^, ^16^, ^17^, ^18^, ^19^, ^20^

The main objective of this study is to compare the agreement between examiners from different specialties and between the same professionals at different times, in relation to the interpretation of radiological signs found in patients with suspected or confirmed NEC.

## Methods

Cross-sectional study for concordance analysis, with voluntary participation of medical specialists who independently evaluated abdominal radiographs of patients suspected or confirmed for NEC, admitted to the Neonatal Intensive Care Unit of the Hospital das Clínicas of the Medical School of Botucatu, from June 2012 to July 2020. Two neonatologists, two pediatric surgeons, and two radiologists participated, all with similar experience in their respective areas of expertise and without direct involvement with the research.

The authors selected for this study plain radiographs of the anteroposterior (AP) view of the abdomen of patients with NEC (at any stage of the modified Bell criteria),^13^ regardless of BW and GA, that were performed no later than 24 h after the diagnostic suspicion. Radiographs of patients with NEC associated with congenital malformations of the GIT and with technical limitations that would impair radiological analyses were excluded.

For the analysis of agreement between the participants, each of the examiners evaluated the radiological images via a form containing the following questions related to the main findings of NEC: the presence of distension of intestinal loops (diffuse or focal), air-fluid level, thickening of the intestinal wall, intestinal pneumatosis, portal venous gas, pneumoperitoneum, and ascites. For the evaluation of intestinal loop distention, the examiner, in addition to subjectively answering “yes” or “no,” also performed measurements of the diameter of the most distended loop (DL), the width of the first lumbar vertebral body (L1), and the distance between the upper edge of L1 and the lower edge of the second lumbar vertebra (L2). These measurements were performed using a millimeter ruler and following the proposal of Edwards et al.^21^ The participants did not have access to information on the clinical conditions of the patients. For the intraexaminer analysis, a new evaluation of the images was performed after two months by one examiner from each specialty (a neonatologist, a pediatric surgeon, and a radiologist). The material provided to examiners is available as supplementary material 2.

The agreement values between different examiners (interexaminer agreement) and between the same examiner at different times (intraexaminer agreement) were determined.

The sample size for the analysis of interexaminer agreement was estimated according to the highest intraexaminer agreement value of 47 % for the identification of intestinal pneumatosis in plain abdominal radiographs of patients with NEC, reported by Rehan et al.^17^ Considering a kappa value of 0.60, with a test power of 90 %, to detect differences of 90 % between the groups, the estimated number of radiographs was 75.^22^ The agreement values were determined by kappa statistics for dichotomous variables, kappa with quadratic weights (Fleiss-Kohen) for ordinal variables, and by the intraclass correlation coefficient for continuous numerical variables. The interpretation of the magnitude of the agreement estimators occurred according to the classification proposed by Landis & Koch.^23^ The proportions of the results obtained by the examiner for each of the forms of interpretation of the plain radiography of the abdomen were compared by means of the binomial test. All analyses were performed with the SPSS v. 22.0 software, considering a 5 % significance level.

The measurements of the DL/L1 and DL/L1-L2 ratios are presented as median and interquartile (IQR) range values. The comparisons of these measurements between suspected and confirmed cases of NEC were performed using the Mann-Whitney method.

The study was approved by the Research Ethics Committee of the Institution (CAAE: 35430220.4.0000.5411). The participants were invited to participate in the study, voluntarily, and signed the Informed Consent Form.

## Results

During the evaluated period, 96 NB were identified with the diagnosis of NEC, which corresponded to 0.56 % of the live births. A total of 115 abdominal radiographs of these patients were performed. After applying the exclusion criteria, a total of 90 AP radiographs were obtained for analysis. The sample consisted predominantly of premature NB (94 %), and 54 % of the patients had the diagnosis of suspected NEC (stages IA and IB) at the time of the radiography (Fig. 1 Sample selection - available as a supplementary material 1). Table 1 describes the main characteristics of the patients.

**Table 1:** Characteristics of the patient sample.

Characteristic	Total *n* = 72
Sex	
Female (%)	31 (43)
Male (%)	41 (57)
Gestational age (weeks)	
≥ 37 (%)	4 (6)
34–36 (%)	13 (18)
32–33 (%)	8 (11)
28–31 (%)	34 (47)
< 28 (%)	13 (18)
Type of delivery	
Normal (%)	26 (36)
Cesarean section (%)	45 (62)
Nutritional status	
AGA (%)	48 (66)
SGA (%)	23 (32)
NEC classification	
I A or B (%)	39 (54)
II A or B (%)	25 (35)
III A or B (%)	8 (11)
BW(g) – (mean ± SD)	1373 ± 604
Age at diagnosis (dl) – (median; P25-75)	6 (3–14)
Abdominal surgery (%)	23 (32)
Deaths (%)	10 (14)

AGA, appropriate for gestational age; SGA, small for gestational age; BW, birth weight; SD, standard deviation; dl, Days of life.

Table 2 shows the results of the analysis of agreement between the three specialties, combined into pairs. The agreement was low (kappa < 0.4) in 30 % of the responses between the neonatologist and the surgeon, and in 38 % between the neonatologist and the radiologist. The highest frequency of low agreement occurred between the surgeon and the radiologist (46 %). Regarding the diagnosis of intestinal loop distention, the greatest agreement between the specialties occurred when the analysis was made objectively when compared to the subjective evaluation of distention.

**Table 2:** Kappa values for interexaminer agreement (Neo × SU, Neo × RD, SU × RD).

Questions (*n* = 13)	Kappa Values		
	Neo × SU	Neo × RD	SU × RD
Intestinal loop distention	0.553	0.489	0.220
If yes, focal or fuzzy	0.180	0.369	–
DL/L1 ratio	0.633	0.778	0.455
DL/L1-L2 ratio	0.840	0.853	0.764
Air-fluid level	0.257	–	–
Thickening of the heart wall	0.367	–	0.175
Intestinal pneumatosis	0.527	0.422	0.349
Gas in the portal venous system	0.522	0.876	0.432
Pneumoperitoneum	0.709	0.788	0.903
Free fluid in the abdominal cavity	0.496	0.345	0.487
If yes, S or M or L	0.692	0.533	0.739
A lot, a little, or not suggestive for NEC	0.233	0.326	0.221
If suggestive, modified Bell rating	0.504	0.480	0.413

Neo, neonatologist; SU, pediatric surgeon; RD, radiologist; DL/L1, ratio of DL diameter to distance from L1; DL/L1-L2, ratio between DL and distance L1-L2; S, small; M, moderate; L, large; (-), null agreement.

Table 3 shows the results for the comparison of the interexaminer agreement between peers of the same specialty. The agreement was low (kappa < 0.40) in 54 % of the answers among neonatologists, 46 % among surgeons, and 85 % among radiologists.

**Table 3:** Kappa values for interexaminer agreement between pairs of the same specialty (Neo, SU, and RD).

Questions (*n* = 13)	Kappa Values		
	Neo *N* = 2	SU *N* = 2	RD *N* = 2
Intestinal loop distention	0.689	0.793	0.135
If yes, focal or fuzzy	0.269	0.178	–
DL/L1 ratio	0.761	0.857	0.829
DL/L1-L2 ratio	0.710	0.533	0.786
Air-fluid level	0.215	0.491	–
Thickening of the heart wall	0.352	0.579	0.173
Intestinal pneumatosis	0.517	0.390	0.245
Gas in the portal venous system	0.645	0.710	0.339
Pneumoperitoneum	–	0.313	0.347
Free fluid in the abdominal cavity	0.129	0.395	0.185
If yes, S or M or L	–	0.469	–
A lot, a little, or not suggestive for NEC	0.517	0.229	0.076
If suggestive, modified Bell rating	0.331	0.204	0.297

Neo, neonatologist; SU, pediatric surgeon; RD, radiologist; DL/L1, ratio of diameter DL to distance from L1; DL/L1-L2, ratio between DL and distance L1-L2; S, small; M, moderate; L, large; (-), null agreement.

Table 4 shows the interexaminer agreement between specialties, comparing suspected cases with confirmed cases of NEC. The degree of agreement was higher in the confirmed cases. In the comparative analyses between the neonatologist and the pediatric surgeon, the concordance category improved in 60 % of the evaluations. Between the neonatologist and radiologist and between the surgeon and the radiologist, this category change occurred in 30 % and 40 % of the evaluations, respectively. Kappa < 0.4 were considered of low agreement. The detection of pneumoperitoneum demonstrated 100 % concordance among suspected cases (absolute agreement to identify the absence of this radiological finding).

**Table 4:** Kappa values for interexaminer agreement between specialties comparing suspected cases of NEC with confirmed cases.

Questions	Kappa Values							
	Neo × SU		Neo × RD				SU × RD	
	SC *N* = 44	CC *N* = 46		SC *N* = 44	CC *N* = 46	SC *N* = 44		CC *N* = 46
Intestinal loop distention	0.218	0.776		0.462	0.551	0.071		0.389
If yes, focal or fuzzy	0.301	–		0.201	0.637	–		–
DL/L1 ratio	0.554	0.723		0.775	0.781	0.422		0.511
DL/L1-L2 ratio	0.757	0.933		0.838	0.865	0.686		0.837
Air-fluid level	–	0.367		–	–	–		–
Thickening of the heart wall	–	0.444		0.018	0.107	0.295		0.542
Intestinal pneumatosis	–	0.642		–	0.429	–		0.498
Gas in the portal venous system	–	0.497		–	0.862	–		0.401
Pneumoperitoneum	1.0	0.631		1.0	0.726	1.0		0.877
Free fluid in the abdominal cavity	0.535	0.474		0.377	0.283	0.482		0.498

Neo, neonatologist; SU, pediatric surgeon; RD, radiologist; SC, suspected cases; CC, confirmed cases; DL/L1, ratio of diameter DL to distance from L1; DL/L1-L2, ratio between DL and distance L1-L2; (-), null agreement.

In the intraexaminer analysis, the neonatologist as well as the pediatric surgeon presented substantial and almost perfect agreement in 12 of 13 responses (92 %). The radiologist, in turn, presented a value of 77 % for substantial and almost perfect agreement. The lowest coefficients of agreement obtained by the specialists in the radiological analyses were the presence of free fluid in the abdominal cavity (kappa: 0.515; by the neonatologist) and thickening of the intestinal wall (kappa: 0.665 and 0.5; by the pediatric surgeon and the radiologist, respectively). The greatest agreement by the neonatologist and the pediatric surgeon occurred in the identification of the radiological finding of gas in the portal venous system (kappa: 1.0 % and 0.921 %, respectively. As for the radiologist, the highest agreement was in the market for air-fluid level, with a kappa of 1.0.

Regarding the diagnosis of bowel distension, concordance was performed by comparing the subjective and objective assessments of the same examiner at different times. The neonatologist's diagnostic agreement was almost perfect in comparisons between the subjective assessment and the DL/L1 ratio measurement (kappa = 0.850) and between the subjective assessment and the DL/L1-L2 ratio (kappa = 0.807). The pediatric surgeon showed substantial agreement in these comparisons (kappa = 0.739 and 0.692, respectively). The radiologist, however, showed low agreement in both comparisons (kappa = 0.261 and 0.378, respectively).

Considering all evaluations, the median (IQR) values of the DL/L1 ratio in confirmed and suspected cases of NEC were, respectively, 1.21 (1–1.5) versus 1.16 (0.93–1.40) (*P* = 0.089). The median (IQR) values of the DL/L1-L2 ratio were 1.13 (0.88–1.4) for suspected cases of NEC, and 1.2 (1.0–1.46) for confirmed cases (*P* = 0.034).

## Discussion

An accurate and early diagnosis of NEC and an appropriate therapeutic indication are crucially important for the prognosis of newborns affected by the disease.^2^, ^3^, ^4^ Despite the limitations in their interpretation, simple abdominal radiographs are, to this day, the most used imaging modality in the evaluation and monitoring of NB with NEC.^15^^,^^18^, ^19^, ^20^

The present study demonstrated the low agreement in the analysis of abdominal radiographs of patients with suspected or confirmed NEC among specialists involved in neonatal care. The authors found a low agreement in 30 % of the answers when comparing between the neonatologist and the surgeon and in 38 % between the neonatologist and the radiologist. The highest frequency of disagreement occurred between the surgeon and the radiologist (46 %). In the calculation for the agreement between peers of the same specialty, neonatologists presented low agreement in 54 %, and the surgeons in 46 % of the evaluations. Among radiologists, low agreement occurred in 85 % of the responses. These results are comparable to those obtained by Markiet et al., who found a low kappa agreement of 0.259 among neonatologists, 0.358 among pediatric radiologists, and 0.274 among radiology residents.^18^ In the publication by El-Kady et al.,^19^ the assessment of agreement showed a reduction in the kappa coefficient when the analysis was performed between different specialties. Among pediatric surgeons, the kappa coefficient was 0.726 and among radiologists, 0.828; however, when comparing surgeons versus radiologists, the coefficient was 0.651.

Notably, the degree of agreement between the examiners was higher in the cases of confirmed NEC compared to the suspected cases. This was observed by the change in the magnitude of the kappa value, especially among neonatologists and surgeons. In this aspect, the pneumoperitoneum marker stands out, of which the agreement was 100 % among the specialists (absolute agreement to identify the absence of this radiological finding). This finding can be attributed to the fact that pneumoperitoneum appears on radiographs as a highly characteristic image of free air within the abdominal cavity. Typically, it is located below and anterior to the diaphragmatic domes or between the liver and the right abdominal wall, making this finding more easily identifiable compared to other images found in NEC.^24^

Several studies have evaluated the role of simple abdominal radiographs in the management of patients with NEC, presenting a low agreement, both intra and interexaminers; however, most did not use a standardized tool for image analysis. Courtney et al., proposed a 10-point scale (Dukes Abdominal Assessment Scale – DAAS) to identify radiological markers and assessed the agreement between four pediatric radiologists.^15^ In this study, the mean intraexaminer kappa value was 0.792 and the mean interexaminer kappa was 0.665. One of the limitations of this study was that it did not define the objective measure of intestinal loop distention.^15^ In this study, the authors applied a specific form with questions about the radiographic markers found in the NEC, and the interviewees measured with a millimeter ruler the parameters necessary for objective evaluation of loop distension (DL/L1 and DL/L1-L2 ratios).

Distention of the intestinal loops is a very common sign in NEC and although it is nonspecific, it is often the first radiographic manifestation and may be related to the severity of the disease.^24^, ^25^, ^26^ The evaluation of distension, in the absence of numerical data, usually uses subjective descriptions, based on the concepts of the evaluator. Obtaining a more objective numerical standard, using specific measurements for analyzing the size of the intestinal loops allows for a more accurate and reliable diagnosis. The limit of normality of the caliber of the intestinal loops has already been published in adults and children; however, due to the great variation in size and weight, there were no defined values for NB. It was only after the publication of a study by Edwards et al.,^21^ in 1980, that the diameters of the intestinal loops were compared to the width of L1 and the distance between L1 and L2.

The present results showed that, when evaluating the intestinal loop distension using objective tools (relation to the DL/L1-L2 ratio), there was an improvement in agreement, with both inter- and intraexaminers. The magnitude of the agreement between neonatologist and surgeon and between neonatologist and radiologist changed from moderate to almost perfect; between surgeon and radiologist changed from low to substantial agreement. In the evaluation among peers of the same specialty, both neonatologists and surgeons presented higher kappa values with the objective evaluation. Among radiologists, the improvement in agreement was more evident, which went from low agreement in the subjective evaluation to a substantial and almost perfect agreement in the objective analyses. These results corroborate the importance of using objective and quantitative methods to define the presence of intestinal loop distention. This parameter can offer diagnostic and prognostic information, with a direct relationship between the measurements of the intestinal loops and the complications of the disease, as well as for the need of surgical intervention and a fatal evolution.^18^^,^^27^^,^^28^ Martins et al.^27^ found that NB with NEC who were submitted to surgery had a 20 % higher DL/L1-L2 ratio than those who did not operate; among those who had complications due to the disease, this ratio was 28 % higher; and among the NB who died, this value was 24 % higher than those who survived (*p* < 0.05 for all comparisons). A similar result was seen in the work of Zvizdic et al.^28^

Regarding the pattern of agreement in the intraexaminer evaluation, the data showed substantial or almost perfect agreement in more than 90 % of the responses of the neonatologist and surgeon and in 77 % of the responses of the radiologist. These data were similar to those found in Courtney's studies, in which radiologists had a mean intraexaminer kappa of 0.792^15^ and both differ from those found by Markiet et al.,^18^ which showed moderate and substantial agreement among radiologists and low agreement in the evaluation of neonatologists.

The statistical method used in the present study was the quantification of the kappa coefficient, which is considered to be the most appropriate and reliable way to evaluate the intra- and interexaminer agreement before a given diagnosis since it is able to correct any results due to chance.^22^^,^^29^

An important point in the intraexaminer analysis concerns the time elapsed between the two analyses. In general, a minimum period of 14 days between the two evaluations is recommended so that the interpretation of the Kappa coefficient does not suffer the so-called “memory” bias.^29^ In the present study, the time interval between evaluations was two months, which reduced the chance of similar responses in the second evaluation due to the effect of the examiner's memory in relation to the first responses.

A limitation of this study would be the small number of participating physicians. However, most studies of agreement analysis use this methodology, considering that the result is more dependent on the number of radiographs evaluated than on the number of evaluators. Notably, in this study, professionals with recognized experience within their specialties were chosen, reducing the chance of interference of this factor in the interpretation of the results.

The present study brings two, thus far, unpublished analyses to the literature. The first refers to the evaluation of agreement comparing suspected cases with confirmed cases, and the second refers to the analysis of intraexaminer agreement in the objective identification of intestinal loop distention, showing the importance of a standardized method based on the use of well-defined measures.

Although it is considered that radiological signs may have a high positive predictive value for the diagnosis of NEC, this study, in accordance with the literature, shows the limitations of its interpretation in clinical practice.^15^ Thus, abdominal ultrasound (US) has been recently used as an important adjuvant for the diagnosis of NEC.^25^^,^^26^^,^^30^ Recent studies by Muchantef and Dilli^20^ emphasize that the two imaging methods, radiography, and US, complement each other and should be used together with the clinical status for the evaluation and management of patients with suspected or confirmed NEC.

Considering that the present study was performed at a single center in a tertiary hospital, the results should be analyzed with caution regarding their generalizability. However, these findings were consistent with those described in the literature, so it is reasonable to consider its external validation.

The data from this study demonstrated low agreement among specialists involved in the management of NEC in this service. The present results underscore the importance of standardizing radiological interpretation, with the adoption of more objective analysis criteria, including the objective assessment of intestinal loop distension through the calculation of the DL/L1 and DL/L1-L2 ratios. This approach aims to improve communication among professionals, optimize diagnosis, and establish appropriate therapy for neonates with confirmed or suspected NEC.

## Ethics approval

The study was approved by the Research Ethics Committee of the Institution (CAAE: 35430220.4.0000.5411). The participants were invited to participate in the study, voluntarily, and signed the Informed Consent Form.

## Conflicts of interest

The authors declare no competing interests.

## Appendix Supplementary materials

Image, application 1
