# Supplementary material for: Analysis of agreement between specialists for the evaluation of radiological findings of necrotizing enterocolitis
Source: J Pediatr (Rio J). 2024 Aug 21;101(1):103–9. doi: 10.1016/j.jped.2024.07.008 (PMC11763538; doi:10.1016/j.jped.2024.07.008)
Supplement: Supplementary file 1 [file mmc1.docx]

**JPED-D-23-00360 – Supplementary Material**

**Supplementary Material**

**NB with suspected or confirmed NEC: 96**

**AP radiographs: 115**

**June 2012 – July 2020**

**Inclusion criteria:**

- Radiographs of NB with suspected or confirmed NEC, 1st image up to 24 hours after suspicion.
- Images in AP – examinations performed in bed.

**Exclusion criteria:**

- NB with GIT malformations: 2 images.
- Radiographs with technical limitations that would impair the visualization of the lumbar vertebrae: 23 images.

**NB: 72**

**AP radiographs: 90**

**Fig. 1**. **Sample selection.**

NB: newborns; NEC: necrotizing enterocolitis; GIT: gastrointestinal tract; AP: anteroposterior.

***Materials Provided to Examiners***

**Instructions:**

- Each image is identified by a number that corresponds to the number on the questionnaire to be completed.
- Measurements: the diameter of the most distended loop DL, diameter L1, and the distance between L1 and L2 should be taken by aligning a ruler with the image on the screen. The adjacent figure serves as an example of how the measurements should be performed.
- The final images are in the left lateral decubitus position and should be analyzed solely for the presence or absence of pneumoperitoneum.

At the conclusion of the instructions, the examiners were provided access to the file containing all the radiological images for assessment.

Below is the questionnaire for the analysis of radiological images.

**Radiological Image Assessment Questionnaire**

**Anteroposterior view**

Distension of intestinal loops? Yes ( ) No ( )

If yes, what characteristic? diffuse ( ) focal ( )

Measurements (cm):

Diameter size of the most distended handle (DL): __ diameter L1: __ distance L1-L2: __

Presence of air hydro level? Yes ( ) No ( )

Intestinal wall thickening? Yes ( ) No ( )

Radiolucency suggestive of pneumatosis intestinalis? Yes ( ) No ( )

Image suggestive of gas in the portal vein? Yes ( ) No ( )

Presence of pneumoperitoneum? Yes ( ) No ( )

Free fluid in the abdominal cavity? Yes ( ) No ( )

If yes: Small ( ) Moderate ( ) Large ( )

**Radiological conclusion**

The analyzed findings are:

Very suggestive of NEC ( )

Not very suggestive, but the NEC hypothesis cannot be ruled out ( )

They do not suggest NEC, and other causes should be investigated ( )

If suggestive, classify according to modified Bell:

Mild (IA, IB, IIA) ( ) Moderate (IIB) ( ) Severe (IIIA, IIIB) ( ).
